# Manufacturing blood *ex vivo*: a futuristic approach to deal with the supply and safety concerns

**DOI:** 10.3389/fcell.2014.00026

**Published:** 2014-06-11

**Authors:** Vimal K. Singh, Abhishek Saini, Kohichiro Tsuji, P. B. Sharma, Ramesh Chandra

**Affiliations:** ^1^Stem Cell Research Laboratory, Department of Biotechnology, Delhi Technological UniversityDelhi, India; ^2^Departments of Pediatric Hematology/Oncology, Research Hospital, The Institute of Medical Science, The University of Tokyo HospitalTokyo, Japan; ^3^Dr B. R. Ambedkar Center for Biomedical Research, University of DelhiDelhi, India

**Keywords:** RBCs, *ex-vivo* erythrocytes, manufacturing blood, hematopoietic stem cells, induced pluripotent stem cells

## Abstract

Blood transfusions are routinely done in every medical regimen and a worldwide established collection, processing/storage centers provide their services for the same. There have been extreme global demands for both raising the current collections and supply of safe/adequate blood due to increasingly demanding population. With, various risks remain associated with the donor derived blood, and a number of post collection blood screening and processing methods put extreme constraints on supply system especially in the underdeveloped countries. A logistic approach to manufacture erythrocytes *ex-vivo* by using modern tissue culture techniques have surfaced in the past few years. There are several reports showing the possibilities of RBCs (and even platelets/neutrophils) expansion under tightly regulated conditions. In fact, *ex vivo* synthesis of the few units of clinical grade RBCs from a single dose of starting material such as umbilical cord blood (CB) has been well established. Similarly, many different sources are also being explored for the same purpose, such as embryonic stem cells, induced pluripotent stem cells. However, the major concerns remain elusive before the manufacture and clinical use of different blood components may be used to successfully replace the present system of donor derived blood transfusion. The most important factor shall include the large scale of RBCs production from each donated unit within a limited time period and cost of their production, both of these issues need to be handled carefully since many of the recipients among developing countries are unable to pay even for the freely available donor derived blood. Anyways, keeping these issues in mind, present article shall be focused on the possibilities of blood production and their use in the near future.

## Introduction

Initially, started by Harvey's studies of blood circulation system, blood transfusion began in the 17th century with animal blood transfusion experiments. The first fully documented report on blood transfusion in humans was from Dr. Jean-Baptiste Denys, who in 1665 successfully transfused blood from a sheep in a 15 years old boy. Though, he could not succeed in later transfusions as recipients died after transfusions were made. In similar studies, Dr. Richard Lower demonstrated the effects of changes in blood volume in circulatory function and developed methods for cross-circulatory study of animals. The first successful human blood transfusion was reported by Dr. James Blundel (1818) between a married couple for a postpartum hemorrhage. However, in 1901, the breakthrough was achieved in human transfusion with the discovery of blood group antigen by Austrian researcher Karl Landsteiner, who discovered that red blood cells got clumped when incompatible blood types were mixed and immunological reaction occurred if the recipient of a blood transfusion had antibodies against the donor blood cells. This “Nobel Prize” (1930) winning discovery made it possible to determine blood type and paved the way for safe blood transfusions. Since then many other blood groups have been discovered. Following to these discoveries a number of blood banks were established during 1940–1950s and it is an inevitable part of all the modern clinical modalities (Alter and Klein, 2008).

The global blood collection was reported to be about 103 million units (www.who.int/worldblooddonorday/en/) (Department of Health and Human Services, 2010, 2013; World Health Organization, 2011). The quality and quantity of donor derived blood collection remain unevenly scattered in economically developed and developing countries. Almost 50% of these blood collections is made in developed countries, which accommodate only a mere 15% fraction of the world's population. Presently, the blood collection seems to be sufficient in economically developed countries. It is supported by reports showing 30,000 annual blood donations on an average per blood center through ~8000 blood centers scattered in 159 high-income countries (Department of Health and Human Services, 2010, 2013). For example, in U.S. the total no. of blood unit collected were 5% more than the actual transfusion made during year 2011 (Department of Health and Human Services, 2013). On the contrary, this number of collections/per center is very less (3700) in developing countries. As per WHO report 82 low income and middle income countries have only 10 donations per 1000 people in the population that would remain highly insufficient to supply a comparable large population residing in these countries (World Health Organization, 2011). Further, the screening facilities are very much inefficient in most of the developing countries. As per WHO record, 39 countries are not able to screen all blood donations for one or more of the following transfusion-transmissible infections (TTIs): HIV, hepatitis B, hepatitis C, and syphilis (Department of Health and Human Services, 2013). Again, there are only 106 countries which have national guidelines on the appropriate clinical use of blood. It would be worth noticing that only 13% of low-income countries have a national haemovigilance system to monitor and improve the safety of the transfusion process. Moreover, the blood supply may look sufficient for the time being in developed countries, it likely becomes inefficient to keep supporting a rapidly growing proportion of elderly population (>60 years age) and burgeoning demand for blood transfusions for surgical treatments by the year of 2050 (U.S. Census Bureau, 2004; Ali et al., 2010).

One of the major challenges in clinical settings is to find blood group compatibility for more than 30 blood group system (308 recognized antigens) including ABO & Rh antigens (Daniels et al., 2009).

There are a number of situations such as rare phenotypes, hemoglobinopathies, polytransfusion patients and polyimmunization that may result in significant complications (World Health Organization, 2011) adding more complexity to the supply constraints. Recent technologies such as antigen masking and enzymatic cleavage to generate universal donor blood groups (O and RhD antigens) have been proposed. But these methods are yet to be developed (Bagnis et al., 2011). While keeping these issues in mind, it is worth noticing that the donated blood is tested for various kinds of infections before any transfusion could be made [e.g., HIV-1, HIV-2, HTLV-1, HTLV-2, Hepatitis B, Hepatitis C, Syphilis (*T pallidum*), Chagas disease (*T cruzi*), and West Nile Virus, Cytomegalovirus (CMV)], and a number of precautionary measures are followed during transfusion procedures. Even then blood transfusions are often associated with several complications (Alter and Klein, 2008; World Health Organization, 2011). These complications not only compromise the quality of treatment, but may add to the overall cost of the regime also which remain associated with it in any clinical setting (Department of Health and Human Services, 2010). This is true even in the developed countries where 0.24 % transfusion has been reported to be associated with adverse reactions (Department of Health and Human Services, 2013). For example, adverse events from transfusions in the US only may account for approximately $17 Billion every year. These complications depend upon the patient status or specific transfusion quantity involved. These situations have evoked researchers to develop alternate methods to get blood from various non-donor derived sources. In the search of blood alternates researchers have developed various artificial molecules mimicking functionalities of blood RBCs. The majority of these molecules is either hemoglobin based oxygen carriers or perfluorocarbon solutions. Hemoglobin based carriers are reported to be inefficient as compared to the oxygen carrying capacity of native RBCs. Whereas, perflorocarbons could not become popular due to their limited functional applications (Winslow, 2006; Henkel-Honke and Oleck, 2007; Natanson et al., 2008; Silverman and Weiskopf, 2009; Castro and Briceno, 2010).

*Ex vivo* expansion of erythrocytes or manufacturing blood from stem cell is the most attracting approach among the global research community for the last few years. The basis for the principle that *ex vivo* cultivated RBCs (or other blood cells) may be of clinical use was provided by Nakamura and his colleagues who have demonstrated that RBCs derived from the immobilized ESCs cell line can protect mice from lethal anemia (Hiroyama et al., 2008). Recently, the proof-of-concept was described by first in-human blood transfusion using *ex-vivo* generated RBCs (Giarratana et al., 2011). In a major landmark study, Giarratana et al. shown the survival capacity of *ex vivo* cultivated RBCs in humans. As described by them, the CD34^+^ cells obtained from volunteer donors as an aphaeresis product were cultured in a serum free media (with the addition of Stem cell factor (SCF), Interleukin-3 (IL-3), and Erythropoietin (EPO) resulting into 2 ml of blood RBCs equivalent (10^10^ cells) under GMP conditions. The *ex vivo* expanded RBCs were labeled with ^51^Cs to trace their fate in patients as approved by FDA U.S. (Giarratana et al., 2011) and demonstrated to be comparable with any native RBCs transfusion. These findings established the proof-of-concept for clinical utilization of *ex vivo* generated RBCs globally. As discussed in the next section, a number of methods have been demonstrated so far (Figure 2), which ensure the feasibilities of *ex vivo* RBCs expansion from various source materials (e.g., CD34^+^ HSCs, Embryonic stem cells, and IPSCs) (Figure 3). There are various factors which essentially regulate the yield and the level of RBCs maturation through these protocols. Reports are there, showing numerical expansion of erythrocyte precursors up to ~0.5 transfusion unit/donation from UCB CD34^+^ cells, and approximately 2 million fold expansions of enucleated erythrocytes functionally comparable to native RBCs through slight modification of culturing techniques (Neildez-Nguyen et al., 2002). Further, large scale production (up to 10 transfusion unit) from two units of UCB have been demonstrated by technically complex protocols (described in later sections) which presently may appear ill suited from the commercial scale production point of view but strengthen the concept that with the advancement of techniques sufficient transfusable units may be produced (Fujimi et al., 2008). Besides that, RBCs precursors, cellular intermediates/nucleated erythroid precursors are generated through these protocols, which may also be of use if transfused. It is expected that these cells are likely to become functionally matured *in vivo* while interacting with internal factors governing their maturation. The same has been reported both in human and animal models (Ende and Ende, 1972; Neildez-Nguyen et al., 2002). Conceptually, the maturing cells along with functionally matured cells may aid to maintain the cellular content at the time of transfusion. These maturing cells may proliferate and each cell may give rise to 4–64 cells before they get enucleated and functionally matured *in vivo*. This hypothesis is supported by the data available from clinical observations in developing countries where 40–80 ml of compatible CB blood (containing 4–7 × 10^10^ RBCs and 2–8 × 10^9^ erythroblast cells) is occasionally used for transfusion in emergent conditions (Ende and Ende, 1972). Together, these reports strengthen the concept that *ex vivo* RBCs expansion likely support the different clinical short-comes to exist due to limited supply and various rare blood based disorder.

However, a number of factors such as technological barriers associated with large scale production and per unit production cost remain to be considered in order to support a large population. The present scale of RBCs production would require to be enhanced several times in a more cost-effective and timely manner. Commercial production of blood may lack essential background financial support in developing countries which are suffering from even more sensitive issues. Moreover, in the absence of more realistic, achievable goals it may become hard even in the capitalistic developed countries. Present article focuses on various issues related to current methods and their feasibilities to realize limited “blood pharming” in near future.

## Erythropoiesis *in vivo*

Hematopoiesis, a dynamic process, is regulated both temporally and spatially. It may be classified as “*primitive hematopoiesis*” in featus where specified cells termed as “blood islands” are generated by yolk sac (YS). In YS, nucleated RBCs exclusively expresses embryonic Hbs (Gower-1, Gower-2, and Hb Portland) are generated for a small period of time. Following to that, a second wave of hematopoiesis or “*definitive hematopoiesis*” produce hematopoietic stem cells, which have the potential of transfusion and includes enucleated RBCs along with various other hematopoietic cells. This event takes place largely in the fetal liver and up to a little extent in YS. In later stages of featus development Definitive hematopoiesis occurs in bone marrow (BM) and that serves as the only site of hematopoiesis in adults through their life. The hematopoietic cells from fetal and adult definitive hematopoietic stages may be differentiated on the basis of their HB contents. In the fetus hematopoiesis α and γ globins, (the components of fetal Hb) (Hb F), are expressed, and in the later β globins (the components of adult-type) (HbA), are expressed (Palis, 2008; Baron et al., 2012).

Erythropoiesis or generation of RBCs includes hematopoietic progenitor cells, which are first differentiated into committed erythroid progenitors and thereafter proliferate/converted to functional red blood cells in a highly regulated hierarchal manner. This dynamic process is tightly regulated through a number of growth factors which ensure the maintenance of regular homeostasis of the RBCs mass.

**Figure 1:**
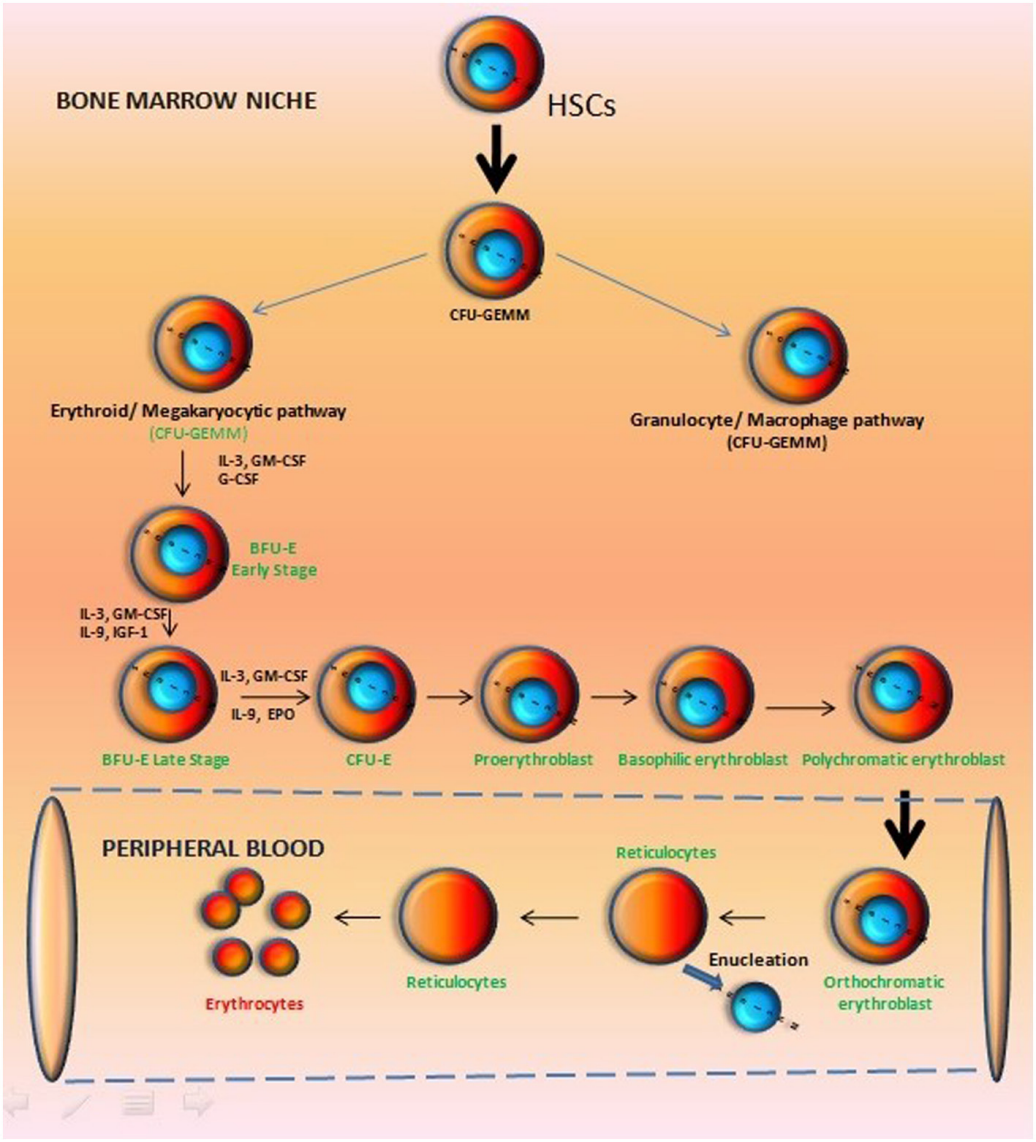
**Hierarchy of erythropoietic development in vertebrates**. In adult Bone Marrow, committed progenitors arising from hematopoietic stem cells give rise to erythroblasts, and as the progeny of a stem cell progress through development, there is a loss of their multipotency while increasing lineage restriction. The various cellular stages in erythrocyte development are identified by their ability to form colonies in semisolid medium supplemented with specific cytokines and by cell surface markers.

The entire process of erythropoiesis may be defined to occur in three stages: (1) Erythropoietic commitment of HSCs: this includes development of erythroid committed blast cells from multipotent hematopoietic progenitors; (2) Division and differentiation of these morphologically identifiable erythroid progenitor cells; and (3) Terminal differentiation including cellular morphological changes such as enucleation, to produce reticulocytes and ultimately maturation: of RBCs (Figure 1).

The Hematopoietic stem cells are generated in YS and may be differentiated into a common myeloid progenitor that, in turn, gives rise to bipotent progenitors restricted to either the granulocyte/macrophage or the erythroid/megakaryocytic pathways (Suda et al., 1983; Debili et al., 1996; McLeod et al., 1996; Akashi et al., 2000). A comparable stage of development defined *in vitro* using cytokine containing semisolid medium (Figure 1) is the colony-forming unit granulocyte, erythroid, macrophage, megakaryocytic (CFU-GEMM) precursor that gives rise to bipotent progenitors restricted to either granulocyte/macrophage or erythroid/megakaryocytic pathways (Suda et al., 1983; Debili et al., 1996; McLeod et al., 1996). Those progenitors which express erythropoietin receptor (EPOR) remain responsive to erythropoietin (EPO) and are committed to the erythroid/megakaryocytic pathway.

On the other hand, EPOR deficient progenitor cells are committed to the myeloid pathway. The burst-forming unit–erythroid (BFU–E), which gives rise to colony-forming unit–erythroid (CFU–E) is termed as the most immature erythroid-restricted progenitor. Among them, early BFU–E (blast-like cells) are highly proliferative, and give rise to clustered burst colonies of up to 20,000 cells in semisolid culture assays.

However, BFU–E bear comparatively small number of EPOR but later progenies (daughter cells) derived from them acquire a high expression level of EPOR and become EPO responsive, transferrin receptor positive, and begin to express hemoglobin (Heath et al., 1976; Lichtman et al., 2007). Later on, CFU–E are derived from these cells, which are highly responsive to EPO, but generate smaller colonies, and express many of the gene products required for definitive erythroid development (Figure 1). There are reports describing a cell division stimulating anti-apoptotic role of EPO in late-stage erythroid cells derived from CFU–E. At this stage of development cells may start the synthesis of hemoglobin and acquire cytoskeletal proteins in tissue culture systems. Moreover, cellular adhesion molecules are expressed in these cells that help define them as nucleated erythroblasts. Thereafter morphologically identifiable nucleated erythroid precursor become visible, which progress from the pro-erythroblast to basophilic, polychromatophilic, and orthochromatic cell forms leading to the reticulocyte (Figure 1) (Stephenson et al., 1971; Lichtman et al., 2007; Jing et al., 2013).

Erythroid differentiation encompasses four distinct major cellular processes such as, (1) the accumulation of hemoglobin that participates in driving the basophilic to acidophilic cytoplasmic changes seen during maturation, (2) limited erythroblast expansion, (3) a continued decrease in cell size, (4) and nuclear condensation leading to their final enucleation. These highly regulated developmental changes take place in the microenvironment of BM niche where “erythroblast islands” are closely associated with macrophages that serve as stromal or nurse cells (Bessis et al., 1978; Manwani and Bieker, 2008; Rhodes et al., 2008). These erythroid development stages end up with the exit of reticulocytes from the marrow, which now enter the circulation and get matured into erythrocytes. The characteristic features of this stage include disassembly of ribosomes, Golgi bodies, and other cellular machinery, removal of organelles, enucleation, changes in the cytoskeleton leading to the classic biconcave discoid shape, and then release into the circulation (Department of Health and Human Services, 2013). Terminal maturation takes place in the erythroblast islands, which involves participation macrophages of with maturing erythrocytes (Bessis et al., 1978; Manwani and Bieker, 2008; Rhodes et al., 2008).

The mature erythrocyte have a life span of 120 days in blood circulation, and afterwards “senescence” occurs reflected through changes in surface antigen expression or physical characteristics of the cell triggering their removal from the blood by macrophages/reticuloendothelial system (Gifford et al., 2006). Thus, hematopoiesis is regulated through changes in body compartments during mammalian development, from embryo to adult, and depending on the age of development, specialized microenvirnmental niches precisely regulate hematopoietic development (Migliaccio et al., 1986; Palis and Yoder, 2001; Baron and Fraser, 2005; Mikkola et al., 2005; McGrath and Palis, 2008).

## *Ex vivo* erythropoiesis: an overview

**Figure 2:**
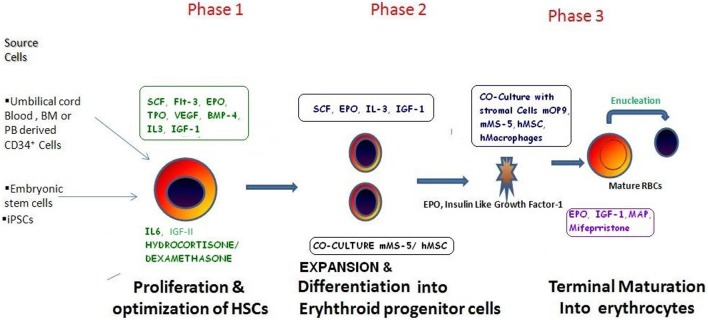
**The various methods/protocols described so far for the *ex vivo* RBCs expansion**. As discussed in the main text, manufacturing blood may involve various step which are to be categorized in three main phases as depicted in the figure. In phase 1: initial source material is to be collected from a variety of source material(s) on the basis of their availability, suitability, and expansion potential and grown in medium generally supplemented with growth factors to enhance HSCs proliferation: subsequently these cells are cultured in the presence of Erythropoietin to induce their differentiation and maintenance into erythropoietic progenitor stage. Finally, in Phase 3 cultures these Erythropoietic progenitors may be co cultured with murine/human stromal cell line support to induce their maturation and enucleation resulting into mature RBCs. These RBCs are evaluated for their biochemical properties and various antigenic profiles to ensure their nativeness.

**Figure 3:**
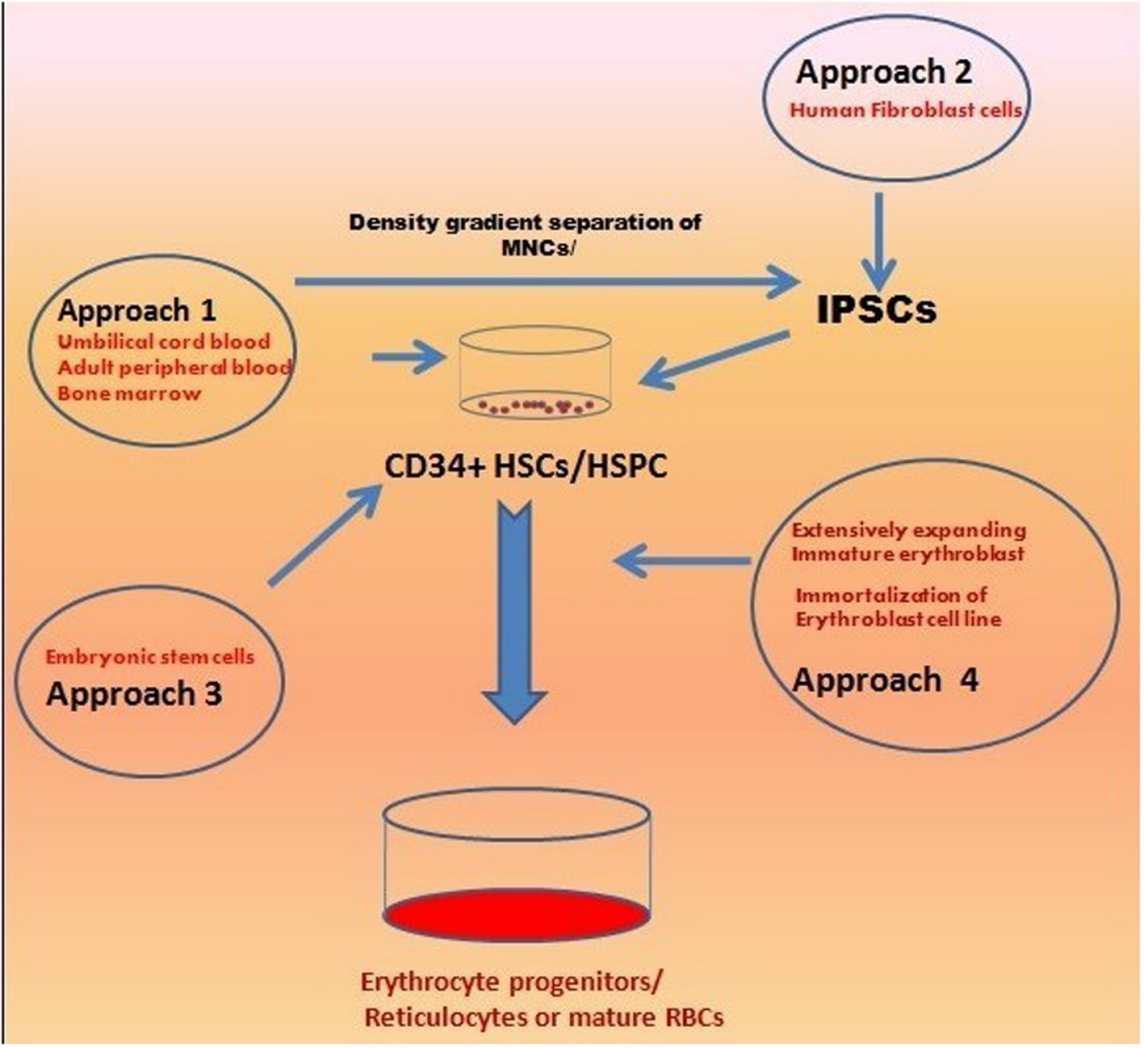
**An overview of the various approaches used for *ex vivo* Erythropoiesis**. The different approaches are in use for develoing large amount of transfusable clinical grade RBCs include CD34^+^ HSPCs (from CB, PB, BM), ESCs/IPSCs derived erythroid progenitors, and highly expanding erythroid progenitors due to stress erythropoiesis. All these approaches has been discussed in detail in text.

There has been considerable progress in the field of developing biological control over the expansion of erythrocytes to generate terminally differentiated, fully functional RBCs (Migliaccio et al., 2012). The *in vivo* hematopoietic development process have been studied (Figure 1) and an in depth knowledge of the generation of erythroblast regulated through various growth factors have paved the way to mimic the process *in vitro* (Lodish et al., 2010). Altogether, these reports demonstrated the enormous potential inherited by three major cell types including (i) CD34^+^ HSPCs, (II) Embryonic stem cells/induced pluripotent stem cells, and (III) immortalized erythroid precursors which can be differentiated into erythroid lineages by using almost similar/overlapping protocols (Figure 2) (Table 1). Regardless of their types, stem cells are promoted to become erythroid progenitor in three basic steps viz. commitment, expansion, and maturation. There are a number of reports showing use of various cytokines/growth factors (SCF and EPO are most common) to generate variable yields of immature progenitors or mature RBCs with or without using animal/human derived feeder coculture system (Figure 2). Recently, a new concept of transdifferentiation (directly differentiating human fibroblast cells into erythroblasts) has been shown to bypass the HSPC state (Figure 3) (Szabo et al., 2010). Although promising, but the technique remains to be evaluated for their potential to generate large scale RBCs. Most of these protocols suffer from similar problems such as degree of enucleation or erythrocyte formation, predominate form of Hb expressed by them, use of animal derived products such as serum and feeder cell coculture, and clinical transfusion related problems such as blood type matching and unavailability of sufficient number of RBCs for transfusion. All these issues can be summarized in the following sections.

**Table 1:** **Progression in the field of *ex vivo* RBCs expansion during last two decades**.

**S. no**.	**Cell source and culture time**	**Growth factors**	**Co-culture**	**Expansion**	**Units of blood**	**Enucleation rate**	**Animal or human serum/plasma**	**Key points**	**References**
1	CB CD34+ 21 days	FLT-3, SCF, TPO, EPO, IGF-1	NO	NA	NA	4% *in vitro*	No	Basis for transfusion potential of *ex vivo* cultured RBCs in animal model	Neildez-Nguyen et al., 2002
						99% *in vivo*			
2	Cord blood 21 days	SCF, IL-3, EPO hydrocortisone	Murine stromal mMS-5	1.95 × 10^6^	4.6/CB	95%	No	First *in vitro* production of RBCs	Giarratana et al., 2005
3	Cord blood 60 days	EPO, SCF, IGF-1 dex and lipid mix	No	10^9^	-NA	−100%	Yes	Prolonged expansion protocol up to 60 days for adult globin switching	Leberbauer et al., 2005
4	Cord blood 20 days	SCF, IL-3, EPO VEGF, IGF-2	No	7.2 × 10^5^	10^4^/CB	77.5%	Yes	Low expansion and enucleation rates	Miharada et al., 2006
5	Cord blood 21 days	SCF, IL-3, EPO, TPO, Flt-3	Human MSCs	8 × 10^3^	0.02/CB	64%	No	Less allogenicity, lower expansion, and enucleation rates, replaced BM derived feeder cells with UCB derived cells	Baek et al., 2008
6	Cord blood 38 days	SCF+Flt-3+TPO IL-3+EPO	hTERT+ Macrophages	3.5 × 10^6^	8.8/CB	100%	Yes	Impossible to scale up	Fujimi et al., 2008
7	hESCs 59 days	Hydrocortisone, IL3, BMP-4, SCF, EPO, IGF-1	hMSCs, mMS-5	4 × 10^7^ cells	NA	(6.5%, starting from CD34+ cells)	Yes	Globin switching	Qiu et al., 2008
			FH-B-hTERT						
8	hESCs 42 days	SCF, Epo, BMP-4, VEGF, bFGF, TPO, FLT3 L	MEFs, OP9	10^10^–10^11^ cells/6-well plate of hESCs	NA	10–65%	No	Functional oxygen carrying capacity of ESCs derived RBCs	Lu et al., 2008
10	hiPSCs,(MR90, FD-136)	SCF,TPO, FLT3-L, TPO, BMP-4; VEGF- IL-3, IL-6		hIPSCs	NA	4–10%	No	First time complete differentiation of hiPSCs cells into definitive erythrocytes capable of maturation up to enucleated RBCs (fetal hemoglobin in a functional tetrameric form)	Lapillonne et al., 2010
	hESCs(H1I) 46 days			4.4 × 10^8^		52–66%			
				^hESCs^ 35 × 10^8^					
11	Cord blood 33 days	SCF, IL-3, EPO hydrocortisone	No	2.25 × 10^8^	−500 units/UCB	>90%	No	First serum-free culture, first demonstration of RBC culture in a large-scale bioreactor	Timmins et al., 2011
12	Cord blood 18 days	SCF, EPO hydrocortisone	No	4.3 × 10^7^	75/CB	70%	Yes	Best yield to date	Giarratana et al., 2011
13	hiPSCs 59 days	Hydrocortisone, IL-3 BMP-4, Flt3L, SCF, EPO, IGF-1	hMSCs/Matrigel.	0.5–8 × 10^6^	NA	NA	Yes	Production of large number of erythroid cells with embryonic and fetal-like characteristics regardless of the age of donor tissue	Chang et al., 2011
			hTERT immortalized fetal liver cells						
14	hESCs hiPSCs 60–125 days	Iron saturated transferrin, dexamethasone, insulin, SCF, EPO, TPO, IL-3, IL-6	MEFs, mOP9 mMS-5	2 × 10^5^ ESCs	NA	2–10%	Yes	RBCs production from transgenic and transgene-free iPSCs using the OP9 coculture method with efficiency comparable to hESCs	Dias et al., 2011
15	hiPSCs 52 days	holo-human transferrin recombinant human insulin heparin, and 5% human plasma SCF, TPO, FLT3 ligand, BMP4, VEGF- n-3, IL-Epo	MEFs	15–28.3 × 10^8^	NA	20–26% RBC and 74–80% orthochromatic erythroblasts	5–10% Hu plasma	First time in a normal and a pathological erythropoietic differentiation models that hiPSC are intrinsically able to mature into adult hemoglobin synthesizing cells	Kobari et al., 2012

### CD34^+^ hematopoietic cells from various sources and their use for *ex vivo* RBCs culture

CD34^+^ cells from peripheral blood (PB), cord blood (CB), and BM have been in use for clinical transplantations for more than two decades (Carmelo et al., 1995). Initialed by Fibach et al. the methods to generate RBCs from CD34^+^ HSPCs have been existing for a long time (Fibach et al., 1989, 1991; Wada et al., 1990). Douay et al. were quick to demonstrate functional characteristics of the erythrocytes generated by CB derived CD34^+^ HSPCs (Douay, 2001; Neildez-Nguyen et al., 2002). Their studies confirmed a higher erythrocyte generating potential in comparison to myeloid precursors with low (4%) enucleation efficiency (Neildez-Nguyen et al., 2002). Giarratana et al. further modified the protocol and demonstrated 90% enucleation efficiency in a similar 3 step method for adult blood/BM derived CD34^+^ cells (Giarratana et al., 2005) by co-culturing them with murine stromal cell line (MS5) or Human mesenchymal stem cells (HuMSC). The procedure resulted in, 20,000 fold, 30000 fold and 2 × 10^5^ fold increment in the erythrocytic cell generation when BM, GM-CSF mobilized PB, and CB derived CD34^+^ cells were used respectively (Douay and Andreu, 2007; Douay and Giarratana, 2009; Douay et al., 2009). This group further reported 5–10 units of blood cells on prolonged culture with feeder cell coculture system (Douay and Andreu, 2007; Douay and Giarratana, 2009; Douay et al., 2009). The cells were reported to have native RBCs like functional characteristics as described by normal glucose-6-phosphate dehydrogenase (G6PD) and Pyruvate Kinase (PK) enzyme levels, membrane deformability and oxygen dissociation characteristics. This was also evident by their similar survival rate in non-obese diabetic/severe combined immunodeficient (NOD/SCID) mice following to intraperitoneal infusion of carboxyfluorescein diacetate succinidyl acetate (CFSE) labeled cells.

These methods suffer from two major drawbacks such as (i) use of animal serum and co-culture system and (ii) low yield of functionally matured RBCs per unit of blood cells, which tempted researchers to identify more defined cocktail of growth factors to achieve a better control over erythrocytes proliferation and differentiation. Malik et al. demonstrated generation of ~10–40% enucleated erythrocytes by enriched CD34^+^ cells from CB, PB, and BM (Malik et al., 1998) in a serum free liquid culture system (with Flt-3, GM-CSF, EPO). This report indicated the possibilities of generating erythrocytic progenitors in the absence of animal derived serum that is strongly recommended by most clinicians. Following to these reports 10^5^ − 1.5 × 10^8^ folds erythroid cell expansion have been reported in similar serum free environment. (Panzenböck et al., 1998; Freyssinier et al., 1999).

Fujimi et al. demonstrated massive production of terminally matured erythrocytes from CD34^+^ CB cells in the serum free environment while avoiding xenogenic feeder by using hTERT transduced human stromal cell line (Fujimi et al., 2008). These methods could result in massive growth of 6 × 10^12^ RBCs from one unit of CB and also the highest RBC number per starting cell (3.52 × 10^6^) in any static culture. However, use of the AB blood group, serum to derive macrophages in a 14 days culture and an additional step of magnetic separation leaves this method ill-suited for the scaling up purposes. Beak et al. showed up to 64% enucleation efficiency by using CB or BM derived mesenchymal stem cells as feeder layer which would require an additional MSCs donor for the feeder cells and thus may not be suitable for large scale production procedures (Baek et al., 2008). Xi et al. has demonstrated an *in vitro* model of human erythropoiesis to study the erythroid differentiation in normal and pathological conditions (Xi et al., 2013). CD34^+^ cells were shown to undergo up to 35 doublings in a prolonged 50 days culture in serum free media supplemented with EPO, synthetic glucocorticoid Dex, IGF-1, SCF, and iron saturated human transferrin. In a subsequent step, matured RBCs could be generated by coculturing erythroid progenitors with human fetal liver stromal cells. The procedure resulted in 10^9^ fold expansion of erythrocytes (Xi et al., 2013).

Recent findings have indicated a significant erythropoietic potential in the CD34neg cell fraction which should also be exploited to enhance the production (Leberbauer et al., 2005; Van den Akker et al., 2010; Tirelli et al., 2011a). Leberbauer et al. described a culture method which exploited the signal transduction cascades of stress erythropoiesis, achieving a 10^9^-fold expansion of erythroblasts of CB cells without prior CD34+ isolation (Leberbauer et al., 2005). Akker et al. describe a culture method modified from Leberbauer et al, and obtained a homogenous population of erythroblasts from peripheral blood mononuclear cells (PBMC) without prior purification of CD34+ cells. This pure population of immature erythroblasts can be expanded to obtain 4 × 10^8^ erythroblasts from 1 × 10^8^ PBMC after 13–14 days in culture. Upon synchronizing differentiation, high levels of enucleation (80–90%) and low levels of cell death (<10%) are achieved. These studies indicated CD34^Neg^ cells as most significant early erythroid progenitor population (Van den Akker et al., 2010). Tirelli et al. also demonstrated significant enhancement (log scale) in the erythroid cell generation by using total MNC in Human Erythroid Massive Culture (HEMA) (Tirelli et al., 2011a). The careful phenotypes examination showed a significantly more erythropoietic potential of CD34^neg^ population as compared to CD34^+^ cells (Tirelli et al., 2011a).

One of the major criteria for calculating efficiency of various *ex vivo* expansion protocols remains to describe the degree of enucleation of these maturing erythrocytic cells. The interactions with macrophages/stromal cells play a crucial role in developing high degree of enucleated erythrocytes as described through various researchers (Kawano et al., 2003). However, most of the stromal cells support used by various researchers are of animal origin and may conflict with the interests of the few clinicians to describe these cells as clinical grade blood products. These issues raised the interest of the research community to define methods without a need for animal based products. Miharada et al. who showed generation of 4.5 × 10^12^ erythroid cells from 5 × 10^6^ CD34^+^ cells in the absence of any feeder cell layer or macrophage cells by using VEGF-1, TGF-1 and a glucocorticoid antagonist Mifepriostone to avoid dependence of these methods on feeder cells (Miharada et al., 2006). Maggakis-Kelemen et al. also reported similar results to improve the yield by using DMSO, Ferrous Citrate and Transferrin in the culture medium (Maggakis-Kelemen et al., 2003). Though these factors significantly increased the yield, but precursor cells shown higher degree of deformability and reticulocytes/erythrocytes were demonstrated to have reduced shear modulus. Beak et al. demonstrated the use of a polymer Poloxamer 188 to yield >95% enucleated RBCs in similar protocols without using any feeder cell support (Baek et al., 2009). Poloxamer 188 enhances the production by protecting cellular lysis during nucleation due to hydrodynamic stress. It provides support to the membranes and protects fragile cells from lysis at the time of enucleation. Thus, it seems that a large number of terminally matured clinical grade RBCs may be obtained regularly with the advent of similar protocol, while introducing newer alternatives at different levels of their expansion. Earlier, similar results were reported by Timmins et al. who demonstrated feeder free culture system by using a bioreactor and resulted in a massive 500 units (10^8^ fold) per UCB donations (Timmins et al., 2011).

While considering these findings, it seems that both CB and PB derived CD34^+^ cells possess enormous expansion potential with some marginal differences such as the predominant form of Hb expressed and % yield of enucleated RBCs. For example, RBCs from CB derived cells are predominantly rich in fetal Hb (Neildez-Nguyen et al., 2002; Giarratana et al., 2005, 2011), whereas, RBCs from PB derived cells majorly express adult Hb (Giarratana et al., 2005, 2011). Apart from that, there have been a significant difference in the expansion potential of CB/PB derived HSPCs. With the current technologies available for their expansion PB derived cells have shown comparatively low (120,000 fold) yields (Giarratana et al., 2005). In contrast, CB derived cells are reported to expand up to 2 million folds. Even more, a single unit of CB derived cells can result into 500 units of transfusable RBCs (Timmins et al., 2011). However, the later method has not been applied for the PB derived cells so far. Thus, it seems that CB shall provide a better option for initial source to be selected under present circumstances, and, if presence of fetal Hb is acceptable (Conley et al., 1963; Weatherall and Clegg, 1975; Thomson et al., 1998; Foeken et al., 2010), it shall remain the most attractive alternate for RBCs expansion protocols. The infrastructure for CB collection is well developed. According to a report in 2010, about 4,50,000 CB units were collected through 131 CB Banks worldwide (Foeken et al., 2010). There is enough information available through these blood bank registries regarding the blood types and combinations of the antigen present on these blood groups for each CB donation. Though, the exact percentage for each rare blood type may not be available and even if the specialized donor could be found for each rare antigen combination, it may be difficult to fulfill a regular demand of transfusion in every 3–4 weeks due to the limited life span of these cells. However, even then a large fraction of the population may be benefitted from the existing technologies without any risk for rare antigen combinations.

### Embryonic stem cells and induced pluripotent stem cells

Embryonic stem cells (Thomson et al., 1998) and induced pluripotent stem cells (Takahashi and Yamanaka, 2006; Takahashi et al., 2007) are defined as immortal cells with the self-renewing capacity and if the requisite growth environment is made available have potential to differentiate into all kinds of cells of the organism. Embryonic stem cells were first isolated from 3–4 days old Blastocysts (Takahashi et al., 2007), and subsequent studies by various research groups described their capabilities to be maintained indefinitely in cultures due to high telomerase activities. Takahashi and Yamanaka's group firstly demonstrated iPSCs or ESC-like cells that may be generated by reprogramming somatic cells. They showed the transformation of murine fibroblast cells in to ESC by transfection of transcription factors such as POUSF-1, KLF-4, SOX-2,and Myc (Takahashi and Yamanaka, 2006; Takahashi et al., 2007). These cells were further demonstrated to form teratoma on infusion in NOD-SCID mice and differentiation into cells from all the three germ cell lines respectively (Okita et al., 2007; Wernig et al., 2007). Generation of Human IPSCs (hips) was also defined by transformation of human neonatal or adult fibroblast by the same set of genes (Takahashi et al., 2007). Yu et al. also generated of hIPSCs from fibroblast cell by using lentivirus mediated transfection of Nanog and LIN28 to reduce the undesired effects of c-Myc (Yu et al., 2007). Subsequently, other methods of generating IPSCs have been reported such as ectopic expression of the same set of transcription factors (Stadtfeld et al., 2008) and use of excisable transposons (Lacoste et al., 2009). Alternatively, these factors could also be provided from outside attached to a cell penetrating peptide sequence (Zhou et al., 2009). Together these cells provide an alternate to generate HSPC. The mononuclear cells (CB/PB) may be reprogrammed in IPSCs and in subsequent procedure human IPSCs could be differentiated into CD34^+^CD45^+^ HSPC by using similar protocols used for ESCs (Ng et al., 2005; Choi et al., 2009).

There are a number of groups who have demonstrated hematopoietic differentiation of hESCs/IPSCs (Lu et al., 2008; Chang et al., 2011; Kobari et al., 2012) (Table 1). These methods can largely be grouped into two categories of (1) Embryoid body formation through suspension culture of hESCs without feeder cells, and (2) co culture of hESCs with murine stromal cell lines such as S17 or OP9 (Chadwick et al., 2003; Vodyanik et al., 2005, 2006; Chang et al., 2006). In the first approach, cells grow in suspension culture resulting in 3-dimensional structured aggregates of differentiating cells called Erythroid Bodies (EBs) (Vodyanik et al., 2005). Chang et al. described a method of generating erythroid cells from embryoid bodies (Vodyanik et al., 2006). Nakamura's group described a method for the generation of enucleated erythrocytes from mouse ESCs (Hiroyama et al., 2008) which was followed by several researchers who demonstrated procedures for the generation of enucleated erythrocytes by using human ESCs (Lu et al., 2008; Qiu et al., 2008; Lapillonne et al., 2010; Dias et al., 2011)

During last 10–11 years significant development has occurred in the procedure to develop hESCs/iPSCs and high yields of approximately up to 10^11^–10^12^ erythrocytic cells have been achieved from a single plate of hESCs (Lu et al., 2008). While comparing these reports, most procedures have indicated an important role of erythropoietin (along with other growth factors BMP-4, VEGF, bFGF etc.) and coculturing them with a feeder cell to derive sufficient yield of enucleated RBCs (Kaufman et al., 2001; Keller, 2005; Ng et al., 2005; Ledran et al., 2008; Zambidis et al., 2008). His can be a Co cultured with a number of feeder cells available, including mesenchymal cell Murine stromal cell line S17, OP9 (Vodyanik et al., 2005, 2006). YS endothelial cell lines (Kaufman et al., 2001), cell lines derived from murine AGM stroma, liver or other developmental niche (Ledran et al., 2008) (Murine AGM derived AM20-1B4 cells) are described as best supporting cells providing higher yields.

In order to achieve animal cell free clinical grade blood products efforts have been made by various researchers. It has been described that the use of feeder cell layer derived from human tissue itself e.g., human foreskin derived fibroblast cells support to generate similar results (Amit et al., 2003; Hovatta et al., 2003; Koivisto et al., 2004). Genbacey and his colleagues, has reported expansion of hESCs in serum free conditions by growing them on cells derived from human early gestation placental fibroblast cells or human placental laminin substrates (Genbacev et al., 2005). hESCs can also be grown in serum free and feeder free conditions by using fibronectin, TGF beta 1, bFGF, and leukemia inhibitory factor (Amit et al., 2003). Bouhasara's group demonstrated hESCs culture on hTERT cell line and their subsequent differentiation into erythrocytic cells in suspension culture method (Qiu et al., 2005; Olivier et al., 2006).

However, there have been ambiguities about the role of EPO/feeder layer played in erythrocytic enucleation; their presence in the final step of most protocols seems to remain inevitable. This is evident from the results obtained by growing these cells in the absence of both the above mentioned factors that result to meager 10% enucleating efficiency (Qiu et al., 2008; Dias et al., 2011). Whereas, inclusion of both EPO/feeder cells has been shown to yield remarkable up to 65% enucleation efficiency of these methods (Lu et al., 2008; Lapillonne et al., 2010). Similarly, IPSCS derived erythrocytes have been shown to depend on a feeder layer for their enucleation in final steps of maturation (Lapillonne et al., 2010; Chang et al., 2011; Dias et al., 2011; Kobari et al., 2012). Recently, heparin and insulin have been reported to enhance enucleation efficiency of IPSCs derived erythrocytes in a feeder free system, but that also remains only 26% (Kobari et al., 2012)

Another important factor is the expression of fetal Hb as a predominant form of protein by the erythrocytes generated in almost all these protocols (Lu et al., 2008; Lapillonne et al., 2010; Chang et al., 2011; Dias et al., 2011; Kobari et al., 2012). This is quite obvious because of the embryonic state of development of both ESCs/IPSc and a more primary route of hematopoietic development (indefinitive) likely to yield predominant expression of fetal/embryonic Hb through these protocols. However, researchers have demonstrated the possibilities of selective expression of adult Hb in erythrocytes by either increasing the time length for coculture (Qiu et al., 2008), or enforced transgene expression of RUNX1a to enhance hematopoiesis from human ESCs and iPSCs (Tirelli et al., 2011a) reporting a higher level of β - globin expression in differentiated erythrocytes (Ran et al., 2013).

So far, there has been no significant difference in the erythroid expansion potential of ESCs and IPSC lines (Dias et al., 2011). The findings reported by Douay's group about 7–8 fold higher erythrocytic expansion potential of human H1 ESC line in comparison to the human IPSCs line (IMR90-16) may be attributed to the viral vectors used for deriving IPSC that has been reported to decrease hematopoietic and erythrocytic expansion potential (Feng et al., 2010). Further, epigenetic variation (integration of viral genomes at different loci) associated with IPSCs generating methods could also contribute to these variations. Similarly, altered hematopoietic potential have been reported for various hESCs lines (Chang et al., 2008).

IPSCs may be advantageous in the conditions where no other alternate in present of generating rare blood group such as O^−^Rh^−^ type of blood group. It has been postulated that only three selected human IPSC lines may be sufficient to generate required blood groups for almost 95.5% of patients in France (Peyrard et al., 2011). While, no such ESC line lines with universal blood donor group has been identified so far in the current list of NIH-approved-for-research (Lu et al., 2008). In addition, IPSCs can be helpful to support autologous transfusion which is highly recommended for alloimmune patients. On the other hand, it has promising reports to generate functional RBCs to cure rare hemoglobinopathies such as sickle cell anemia and β-Thalassemia (Hanna et al., 2007; Zou et al., 2011).

### Erythroid precursor from stress erythropoiesis

In addition to the above mentioned stem cells, researchers have shown a significant RBCs generation potential in the extensively proliferating erythroid precursors which are developed due to stress conditions such as erythrolysis or hypoxia (Figure 3). The process, generally termed as “stress erythropoiesis,” relies upon glucocorticoids and their counter receptor interactions. There are reports showing up to 10^10^-fold expansion of CB-derived erythroid precursors using Dexamethasone as a glucocorticoid agonist along with other growth factors, e.g., SCF, EPO, and IGF-1 (Bauer et al., 1999; Von Lindern et al., 1999; Leberbauer et al., 2005). Similar results could be obtained for the PB derived erythroid precursors with the use of IL-3 along with above mentioned cytokines (Migliaccio et al., 2002). However, these approaches were reported to be of limited use due to their minimal fold expansion (<20 fold) which remain insufficient as per clinical transfusion is concerned (Migliaccio et al., 2010).

In contrast, there have been advancements in generating immortalized or extensively expanding erythroblast cell lines from embryonic stem cells with the ability to produce enucleated erythrocytes in mouse (Carotta et al., 2004; Hiroyama et al., 2008). Similar extensive expansion potential has been demonstrated by mouse embryos (England et al., 2011), which indicated a higher proliferation potential of embryonic erythroid progenitors than postnatal cells. It is hypothesized that if postnatal erythroid cells could be reprogrammed by using existing techniques to the state of embryonic erythroid progenitor cells they could also proliferate up to a similar extent. In fact, it has been demonstrated by Cheng's group (Huang et al., 2013), who has demonstrated immortalization of human CB-derived erythroblast by using similar reprogramming factors defined by Yamasaki's group (Takahashi and Yamanaka, 2006). The Author has demonstrated a significant 10^68^-fold expansion of CB-derived erythroblasts (in~12 months) in a serum-free culture. The ectopic expression of three genetic factors Sox2, c-Myc, and a shRNA against TP53 gene enabled these cells to undergo an expanded expansion in similar culture condition. These cells showed normal erythroblast phenotypes and morphology, a normal diploid karyotype and dependence on a specific combination of growth factors for proliferation throughout the expansion period. The coculture of these cells with irradiated mouse OP9 cell line in the presence of serum yield to 30% enucleation efficiency with increased fetal Hb expression level. Similarly, Kurita et al, demonstrated immortalization of human IPSC or CB derived cells by inducible expression of TAL-1 and HPV-16-E6/E7 viral gene (Kurita et al., 2013). Thus, it is expected that with more advancements in the reprogramming technology such as the use of nonviral episomal plasmids, synthetic RNA, and small molecules, development of more donor cell line would facilitate generation of all blood types (Chou et al., 2011; Hou et al., 2013; Yoshioka et al., 2013).

## *Ex-vivo* culturing of other blood components

### Platelets

Platelets are small anuleated, discoid shaped, 1–2 μm cell fragments that play essential role in the maintenance of homeostasis, stopping bleeding, or wound healing (thrombosis), inflammation, and innate immunity. There is a burgeoning use of plasma transfusions to control massive bleeding in clinics. The combination of red blood cells, platelet concentrates, and plasma is used for the same Platelets are present in much higher densities in vertebrate blood (5.5 platelets × 10^10^/unit) that are generated from megakaryocytes and their precursor cells in BM (Machlus and Italiano, 2013).

Platelets are generated from megakaryocytic cells in the BM niche under the regulation of various growth factors. Unlike RBCs, committed HPCs can be easily recruited into megakaryocytic lineage differentiation pathways by their induction through a single growth factor TPO. This straight forward mechanism has tempted many researchers to mimic similar differentiation under research settings. Like RBCs, there have been efforts to generate platelets from all different sources, including CD34+ HSPC., ESCs/iPSCs and immature megakaryotic precursor cells. Christian et al. have demonstrated the generation of platelet precursor cells from human CD34^+^ cells by using SCF, TPO, IL-3/IL-11, and Fetal Liver Tyrosine kinase-3 ligand (Flt-3L) in the liquid culture system (Christian et al., 2010). However, these precursors were shown to undergo recirculation in hematopoietic tissues on transfusion in mice. Proulx et al. demonstrated ~300 fold expansion of MK/CD34^+^ by growing UCB derived CD34^+^ cells with SCF, Flt-3L, TPO, and IL-6 at high concentrations (Proulx et al., 2003, 2004). Growth of MK/CD34^+^ may further be improved by optimizing culture conditions such as temp (39°C), O_2_ tension and addition of additional factors such as SDF-1α and Nicotinamide (Guerriero et al., 2001; Mostafa et al., 2001; Cortin et al., 2005; Giamniona et al., 2006). Matsunaga et al. (2006), demonstrated generation of 3.4 unit platelets from UCB-CD34^+^ cells by using hTERT mesenchymal cell coculture method in 33 days (Matsunaga et al., 2006). Sullenberger, also reported a 3D cartridge based perfusion bioreactor with the capacity of continuously producing platelets over a period of 30 days (Sullenharger et al., 2009). Moreover, the efforts described so far for producing platelets from UCB derived cells remain largely insufficient to fulfill even a fraction of global demand.

Recent advancements have demonstrated enormous *in vitro* platelets generation potential in pluripotent stem cells (including iPSCs/ESCs), and a comparatively new type of cells called induced MKs (iMKs) (Masuda et al., 2013). Matsubara et al. showed a direct conversion of fibroblasts (mouse/human) into megakaryocytes (MKs) by using three factors p45NF-E2, Maf G, and Maf K, which subsequently can release platelets (Ono et al., 2012). The authors identified significance of these factors in generating abundant MKs and platelets from human subcutaneous adipose tissues (Matsubara et al., 2009, 2010; Ono et al., 2012).

In another approach, ESCs/IPSC mediated generation of platelets in humans has been reported (Takayama et al., 2008, 2010). Eto et al. showed that c-Myc expression (essential for reprogramming of IPSCs) must be reactivated transiently and then shut-off (Takayama et al., 2010) and to ensure efficient platelet production from human IPSCs. This transient expression might be essential because continuous excessive c-Myc expression in IPSCs derived MKs was shown to increase p14 (ARF) and p16 (INK4A) expression, and decreased GATA1 and NF-E2 expression, which eventually resulted MK senescence and apoptosis, as well as impaired production of functional platelets (Takayama et al., 2010).

More recently, Eto and colleagues have established an immortalized MK cell line (MKCL) derived from human IPSCs (Masuda et al., 2013). The immortalized MKCL could potentially provide a stable supply of high quality platelets for transfusion medicine. However, this method remains elusive and a detailed protocol suitable for commercial production is yet to come.

Thus, recent findings indicate strong possibilities of a regular non donor derived platelet supply in near future. However, there are several factors that shall be discussed to compare existing approaches. While comparing with IPSCs/ESCs based methods, the iMK cells (derived in these studies) are rapidly converted (2 weeks) into MKs cells, but yield remain poor as fewer platelets (5–10 platelets per iMK cell was generated. In contrast, single MKs may give rise to up to 2000 platelets under *in vivo* conditions. Hence, one of the important factors shall remain to be discussed is the total number of cells that can be obtained from each cultural setting. For example, Matsubara et al reported generation of 8–10 × 10^5^ iMKs from 20 × 10^6^ Human Derived Fibroblasts (HDFs) (Ono et al., 2012). HDFs are easily expanded following to their initiation of direct conversion with limited number of transfected cells, whereas, 200–300 IPS clones from 10^5^ HDFs were reported by Eto and colleagues in their studies. Further, 1 × 10^5^ cultured IPSCs gave rise to 17 × 10^5^ MKs (Takayama et al., 2010). Thus, these results indicated low platelet production efficiency per MK in both strategies.

Another important issue is the time taken for the production of MKs and platelets through these protocols. iMKs and iPSC-mediated MKs are induced in approximately 2 weeks and 2 months, respectively. However, it is yet to be established, but MKCLs may be a much faster approach to generate platelet sources for platelet production due to their direct expandability into MKs.

Further IPSC-mediated MKs are not completely free from the possible risk of some residual undifferentiated cells However, a possible approach may be to avoid transfusion of MKs while preferring fully matured platelets derived from these methods. Platelets (anucleated cells) may be irradiated before transfusion and thus may become free of any contamination of residual undifferentiated cells.

IPSCs and MKCLs are immortal cell lines and thus are comparatively easy to freeze down. Similarly, HDFs are also suitable for cryopreservationm from where they can be taken out and used as starting materials for iMK induction. HDFs in comparison to platelets show better suitability for cryopreservation. The functionality of iMKs has been assessed through their infusion into irradiated immunodeficient NOD/Shi-scid/IL-2Rγ^null^ (NOG) mice showing normal release of human platelets *in vivo* (5–10 platelets per infused IMK) cells and equal capabilities of forming a thrombus in *ex vivo* conditions formation under flow condition (Ono et al., 2012).

The iMKs are reported very recently and the early outcomes indicate them as a valuable option for transfusion medicine. However, there are some important issue which shall be considered in much detail such as the molecular mechanism by which iMKs are induced through the p45NF-E2 transcriptional complex, and identification of most suitable cells for direct conversion into iMKs (or for establishment of MKCLs).

One of the important issues to be discussed here is to explore the necessity of generating autologous iMKs through direct conversion for clinical transfusion. Under most clinical settings, platelet transfusion is done without any prior matching of human leukocyte antigen (HLA) between the donor and recipient. However, in rare conditions repeated platelet transfusions can elicit undesired production of anti-HLA antibodies against transfused platelets leading to an inevitable demand to identify HLA-matched platelet donor (Stroncek and Rebulla, 2007). A possible alternate may be provided by using autologous iMKs based strategies. In addition, establishment of iPSC banks shall be an alternative approach to deal with this problem. These banks shall store homozygous HLA-typed iPSCs which would be deposited for HLA-matched tissue transplantation (Taylor et al., 2012). It has been demonstrated that a tissue bank from 150 selected homozygous HLA-typed volunteers could match 93% of the UK population (Taylor et al., 2012). Yamanaka et al has also postulated that 140 unique HLA homozygous donors shall remain sufficient for 90% of the Japanese population (Okita et al., 2011).

### Neutrophils

Neutrophils play important role in cellular immunity, but unlike RBCs and Platelets they are not routinely collected in clinics. Neutrophils are need to be transfused in relatively large amount, i.e., >10^10^ calls every day in neutropenic patients. *Ex vivo* expansion of neutrophils has been reported by some groups showing ~400 fold expansion from single units of UCB-CD34^+^ cells (5 × 10^6^ CD34^+^ cells), which equivalents to a mere 20% of the required daily dose. Although, sufficient cells may be produced by using PB mobilized by cytokines as demonstrated by Dick et al. (2008), who showed ~534 × fold (equivalent to 10 neutrophil units) expansion on *ex vivo* culture of these cells. But this would mean one donor per recipient and supply constraints largely remain unsolved. Similar efforts to generate neutrophils from hESCs have also resulted in very low yields (Saeki et al., 2008). These reports indicated the need for developing more efficient and simple procedures to be developed first for the generation of neutrophils from either UCB-CD34^+^, BM derived cells and/or houses before their commercial manufacturing could be planned.

## Factors regulating the fate of commercial blood manufacture and future prospects

### Source material suitability and availability

Scientists have been trying to develop a consensus on the different variables controlling the fate of *ex vivo* RBCs expansion and clinical use. The most important of them is the source materials for various methods being explored to expand RBCs in large scale. Ideally, a source material should be a discarded material so that no extra cost would be aided in the commercial production process. At the same time, if a regular supply chain of *ex vivo* manufactured blood has to be maintained the source material would also be required on a regular basis i.e., unlimited availability of the source material should be ensured. The most important factor is the capability of the source cells to get developed into the finally matured RBCs cells with absolute efficiency without raising any immune response in the host i.e., it should be non-immunogenic. The criterion fits well with umbilical CB, which is available abundantly and is a waste product in all the maternity hospitals. In fact, a significant number of transfusion can be made for the patients who are suffering from rare hemoglobinopathies (~ 1%) by using *ex vivo* cultivated RBCs units that might be obtained from these sub threshold units of CB (>90 ml) discarded regularly. Each discarded unit of CB may generate 10–75 RBCs products which might be useful in transfusions for rare hemoglobinopathies (Peyrard et al., 2011). Similarly, Leukopheresis is routinely done in clinical centers where transfusions are made to treat various leukemia patients. In these protocols the leuko-reduction buffy coat is produced as a byproduct and this contains significant amounts of RBCs producing cells (equivalent to umbilical CB) (Zhou et al., 2009). The proof-of-the concept that these *ex-vivo* cultivated cells may be of clinical use was provided by demonstrating successful transfusion of CD34^+^ cells which were obtained from a G-CSF mobilized donor (Migliaccio et al., 2010). Apart from UCB derived CD34^+^ cells, ESCs, and hiPSCs as described in previous sections might serve as a potential source for regular manufacturing processes with the advancement in technologies.

### Quantitative issues

The most frequently transfused blood component, RBCs, are present in 2 × 10^12^ cells/unit (200 ml of blood) with an expected cell density of 3 × 10^6^ cells/ml. According to the WHO data >100 million units of blood is collected every year worldwide. In order to replace this huge volume with synthetic blood/blood components, an extremely large amount of ingredients along with skilled manpower would be an inevitable requirement. It is hypothesized that a cell density of 5 × 10^7^ cells/ml would be essential to produce regularly to support this much demand. Further, according to an assumption, manufacturing of a single unit of blood by using present static culture methods, would require 660 liters of culturing medium and 9500 lab scale 175 cm^2^ culturing flasks (70 ml medium/flask) (Timmins and Nielsen, 2009; Zeuner et al., 2012).

Ensuring such a high concentration of RBCs would attract development of more automated methods like bioreactors that might be helpful in reducing the culturing assets and associated labor. Fortunately a wealth of information regarding the use of various types of bioreactors for HSCs expansion has been accumulating during the last two decades. This could help researchers achieve required cell density. It was reported by Timmins et al that similar density of RBCs as in static culture can be produced in a wave bioreactor (Timmins et al., 2011). A maximum cell density of 10^7^ cells/ml was also reported in a stirred small scale bioreactor (Ratcliffe et al., 2012). Housler et al, reported a massive yield of 2 × 10^8^ cells/ml in a hollow fiber bioreactor (Housler et al., 2012). These 3D bioreactors can be scaled up with current design to produce 1–2 units of RBCs in 3–4 weeks of time. These reports indicate possibilities of ensuring a regular production of large amounts of RBCs, however, a number of factors such as degree of cell maturation and various cultural parameters are yet to be defined in a more elaborated manner.

Further, an important achievement could be the development of methods to commit the source material directly into the erythropoietic lineages reducing both the time and cost of production. There are few preliminary reports about various efforts to define alternate sources such as (i) generation of erythropoietic cell lines from ESCs/IPSCc (i) reprogramming any somatic cells directly into erythroblasts by passing the pluripotent state through over expression of suitable genes.

This has been reported for megakaryocytic differentiation also where human fibroblast cells are directed to differentiate directly into megakaryocytic cells through over expression of p45NF and E2/MaF transcription factors (England et al., 2011; Wang et al., 2011). Cheng's group reported in humans the differentiated erythroid cells obtained from human embryo might be used as a potential initial source for RBCs production (Huang et al., 2013; Kurita et al., 2013). Thus, it is conceivable that with more advancements in the culture methods and initial source material a significant amount of RBCs could be produced for limited clinical uses.

### Growth factors

Growth factors used in various phases of *ex vivo* culturing should also be explored to regulate the fate of commercial manufacturing. Both the “*concentration*” and “*time*” of their administration would play an important role in the optimization of the expansion process. It is also to be noticed that there are various growth factors which express gene polymorphism, for example, >260 isoforms of human glucocorticoids receptors are expressed which are likely to differ in their potency and effects. In order to secure a large scale production of RBCs, it is conceivable to determine an in depth knowledge about the functioning and efficiency of various isoforms through high throughput screening methods regularly. A good example might be the use of reverse phase proteomic analysis of CD34^+^ cells to define molecular mechanism of signaling pathways activated during erythroid differentiation. Recently, similar studies have been reported by various groups showing activation of different set of gene(s) in CD34^+^ cells and erythroblasts. Genes that are involved in the inhibition of apoptosis, transcription, and proliferation are more active in CD34^+^ cells, whereas erythroblasts predominantly express those genes which play important roles in regulation of cell cycle, transcription and translation (Ratcliffe et al., 2012). This information may be of use if specific pharmacological agents enhancing erythrocytic pathways and blocking apoptotic pathways may be introduced at various phases of *ex vivo* expansion.

### Biophysical and biochemical parameters

Generation of clinical grade RBCs would require development of stringent quality checks for *ex vivo* expanded RBCs. The major parameters would include determination of antigenic profiles, hemoglobin contents and physical progenies derived from these culture processes. As described by various laboratories *ex vivo* generated RBCs are slightly macrocytic but normal in size. These RBCs also expresses a great level of α-hemoglobin stabilizing proteins (AHSP), BCL11A and globin gene with great donor to donor heterogeneity (Keel et al., 2008). It is also reported that *ex vivo* generated erythroblasts contain γ-globin in a slightly greater amount in comparison to RBCS which are generated *in vivo* (0.12–0.20 pg/cell with respect to total protein content of 18.723.7 per cell) (Lu et al., 2008).

### Antigenic profile

Surface antigens are vital for the function of RBCs and a large number of surface antigen have been identified on the surface of mature RBCs. *Ex vivo* generated RBCs are reported to express normal level of antigens present on both the Ankyrin A (GPA, M/N, Ena FS, RhAO, and band 3/4.1 R) and Glycophorin B complex, the urea transporter, the complement receptor, and receptors that protect RBCs from complement mediated lysis Recent reports about the surface antigenic analysis of the *ex vivo* generated RBCs ensured that appropriate level of surface antigen such as band 3 and Rh antigen is assured at the beginning of the maturation process (Satchwell et al., 2011).

### Ethnicity

Recently, there have been some reports demonstrating effects of ethnicity on the source material used for *ex vivo* expansion. African-American donor derived MNC were found to be found to be more useful as per as fold expansion is concerned since they could be expanded up to 14–40 fold in comparison to the Caucasian population derived MNCs which give rise to 1.7–30 fold expansion (Tirelli et al., 2011b; Carilli et al., 2012)

### Enucleation of RBCs

Since, *Ex vivo* expanded RBCs shall essentially be evaluated for their degree of terminal maturation in all the production units. Hence, high level of enucleation of maturing erythroblast is an inevitable requirement. Erythroblasts derived from ESCs are demonstrated to yield >60% enucleated RBCs (Lu et al., 2008) while the nucleation efficiency of maturing erythroblast from ups could be demonstrated to a mere 4–10% of RBCs produced (Lapillonne et al., 2010). Recently, there have been efforts to define the factors regulating enucleation efficiency of RBCs indicating the significance of proteins such as Huston deacetylase (specifically HDAC-2 isoform) which are required for chromatin condensation and enucleation in mice fetal erythroblast (Chang et al., 2011). Since, glucocorticoids play important negative regulators for these proteins as mentioned above, application of molecular agents which specifically activates HDACs could be employed in phase-3 cultures to enhance the enucleation yields. As mentioned in a number of reports, co -culturing of maturating erythroblast along with stromal cells may enhance their terminal maturation, but on the other hand it might impose an additional risk of poorly defined potentially immunogenic and/or infectious agent which may compromise the production of GMP for RBCs in the developmental process. These issues indicate the need for the development of biochemical alternatives to avoid the use of stromal co-cultures in these protocols. There are preliminary reports on the use of drugs like Mifepristone and plasmanate which are defined to have similar effects on promoting enucleation efficiency during *ex vivo* expansion of RBCs (Miharada et al., 2006). In addition, factors promoting the functioning of proteins involved in vesicle trafficking such as “vaccuoline” may also be used to improve the enucleation efficiency if phase-3 cultures (Keerthivasan et al., 2010).

### Production cost

The regular commercial production of RBCs would be heavily affected by their cost of production. According to a hypothetical estimation, producing RBCs in desired range would cost approximately $8000–15000 per unit (Timmins and Nielsen, 2009; Zeuner et al., 2012). Whereas, the hospital cost for leuco-reduced RBCs is only $225 that is significantly lower in comparison to synthetic product. There are situations such as matching of rare phenotypes of RBCs and the present cost of a phenotypically matched unit of RBCs is ~$700–1200. Producing blood on a commercial scale would require a huge monetary investment and it will be difficult to keep the momentum in the direction of R&D for developing more and more efficient protocols due to lack of money especially in low income grade countries. It would be important to find out immediate goals which can be achieved from the outcomes of the existing research findings to generate alternate sources of money. For example, immediate use of these small scale products may be useful in developing various immediately achievable goals. There are few promising examples, such as the use of *ex vivo* cultured cells for Reagent RBCs diagnostics in specifically alloimmunized patients and they can be used as drug delivery vehicles for personalized therapies. The requirement of relatively small no. of RBCs in these methods makes them most suitable for these kinds of assays, for example, ~2 × 10^8^ RBCs will be sufficient to perform 100× s of these assays in clinical settings and with the present methodologies 10^9^–10^10^ RBCs can be expanded regularly.

## Summary

In brief, it seems feasible that commercial production of RBCs may take place in near future. There are enough technological evidences available supporting the concept and with the advent of newer source materials, optimal methods for *ex vivo* cultivation of RBCs and highly stringent, comprehensive quality control, analytical measures, the regular production of blood may take place to support a smaller fraction of the population at least. All the three major sources of like CD34^+−^ HSCs (UCB, PB, and BM derived cells), hESCs and, iPSCs have enough potential of generating huge amounts of RBCs and if more sophisticated methods are generated such as development of erythropoietic hESC cell lines, it would be possible to generate an unlimited source of initial source material for the production measures.

Moreover, with the development of more in depth knowledge about the molecular mechanisms involved in various stages of *ex vivo* erythropoiesis. It would become more efficient to support even larger population's blood demands. The fundamental information shall also be helpful in developing pharmacological alternates for the various stages of erythropoietic development which presently depend upon their interactions with feeder cell support systems in most of the protocols. Development of pharmacological alternates is essential to simplify the production processes for clinical grade blood. Avoiding the need of feeder cells may also help in reducing the unexpected risks associated with their use due to undefined immunogenic and infectious exposures which are likely imposed to the recipients by animal derived feeder cell support methods. The omission of feeder cells from *ex vivo* RBCs expansion protocols would also simplify the commercial scale production methods and may also be helpful in reducing the cost of production. As per as cost is concerned, a small fraction of the population may be capable of bearing the cost of commercially produced blood in economically developed countries. However, more liberal government policies such as lowering both the sale and services taxes, provisions for subsidized input materials (like electricity, water, etc.), direct subsidy offered for the products may be helpful in supporting the low cost production of blood. Besides that, a small amount of remuneration may also be generated from the various smaller/specific products such as reagent RBCs, RBCs for the specific drug delivery targets to cure rare hemoglobinopathies and that might support the ongoing research and development in the same direction.

### Conflict of interest statement

The authors declare that the research was conducted in the absence of any commercial or financial relationships that could be construed as a potential conflict of interest.
